# Supplementary material for: How sex impacted associations between psychological distress and worry on adults’ health behaviours during SARS-CoV-2
Source: PLoS One. 2025 Dec 29;20(12):e0339274. doi: 10.1371/journal.pone.0339274 (PMC12747350; doi:10.1371/journal.pone.0339274)
Supplement: S2 Table — Odd ratios and 99% confidence intervals shown. Reference group for sex (male), clinical anxiety (no diagnosis), clinical depression (no diagnosis), wave (6). n = number. *p < .01. amoderator p-value < .10. Interaction term kept in multivariable model. (DOCX) [file pone.0339274.s002.docx]

| **Canada** | **Physical activity** | **Diet** | **Alcohol** |
| --- | --- | --- | --- |
| **Model 1** | **n=10,650** | **n=10,913** | **n=8,115** |
| Psychological distress | 1.48 (1.36-1.61)* | 1.78 (1.61-1.98)* | 1.86 (1.67-2.08)* |
| Sex | 1.05 (.87-1.26) | 1.38 (1.07-1.78)* | .83 (.62-1.11) |
| Psychological distress x sex | 1.03 (.92-1.15) | .91 (.79-1.04)^a^ | .97 (.83-1.13) |
| Age | 1.00 (.92-1.15) | .99 (.98-.99)* | .99 (.98-.99)* |
| Clinical anxiety | 1.06 (.90-1.24) | 1.11 (.93-1.33) | 1.05 (.85-1.28) |
| Clinical depression | 1.12 (.94-1.34) | 1.34 (1.11-1.63)* | 1.22 (.98-1.53) |
| Wave 7 | .85 (.73-.99)* | .82 (.68-.97)* | .87 (.71-1.05) |
| Wave 8 | .90 (.77-1.04) | .89 (.75-1.07) | .91 (.74-1.10) |
| Wave 9 | 1.10 (.95-1.27) | .91 (.76-1.08) | .89 (.73-1.09) |
| **Model 2** | **n=10,723** | **n=10,994** | **n=8,159** |
| Worry | .89 (.81-.97)* | .93 (.83-1.04) | .84 (.75-.94)* |
| Sex | 1.25 (.95-1.66) | 1.37 (.98-1.91) | .68 (.46-.98)* |
| Worry x sex | .98 (.86-1.11) | .99 (.85-1.15) | 1.15 (.97-1.36)^a^ |
| Age | 1.00 (.99-1.00)* | .98 (.98-.98)* | .98 (.97-.98)* |
| Clinical anxiety | 1.27 (1.08-1.48)* | 1.40 (1.17-1.67)* | 1.32 (.108-1.62)* |
| Clinical depression | 1.35 (1.13-1.60)* | 1.67 (1.39-2.02)* | 1.56 (1.26-1.93)* |
| Wave 7 | .84 (.72-.97)* | .81 (.68-.96)* | .85 (.70-1.02) |
| Wave 8 | .89 (.77-1.04) | .89 (.75-1.06) | .89 (.73-1.07) |
| Wave 9 | 1.08 (.93-1.25) | .91 (.77-1.08) | .88 (.73-1.07) |
| **Model 3** | **n=10,784** | **n=11,059** | **n=8,199** |
| Sex | 1.22 (1.20-1.36)* | 1.35 (1.19-1.53)* | .92 (.80-1.06) |
| Age | 1.00 (.99-1.00)* | .98 (.98-.98)* | .98 (.97-.98)* |
| Clinical anxiety | 1.28 (1.10-1.50)* | 1.42 (1.19-1.69)* | 1.34 (1.10-1.63)* |
| Clinical depression | 1.36 (1.14-1.61)* | 1.68 (1.39-2.03)* | 1.57 (1.27-1.95)* |
| Wave 7 | .85 (.73-.98)* | .81 (.68-.96)* | .85 (.70-1.03) |
| Wave 8 | .89 (.77-1.03) | .89 (.75-1.05) | .89 (.73-1.07) |
| Wave 9 | 1.08 (.93-1.25) | .91 (.77-1.08) | .88 (.73-1.07) |
