# Supplementary material for: How sex impacted associations between psychological distress and worry on adults’ health behaviours during SARS-CoV-2
Source: PLoS One. 2025 Dec 29;20(12):e0339274. doi: 10.1371/journal.pone.0339274 (PMC12747350; doi:10.1371/journal.pone.0339274)
Supplement: S3 Table — Odd ratios and 99% confidence intervals shown. Reference group for sex (male), clinical anxiety (no diagnosis), clinical depression (no diagnosis), wave (1). n = number. *p < .01. amoderator p-value < .10. Interaction term kept in multivariable model. (DOCX) [file pone.0339274.s003.docx]

| **Columbia** | **Physical activity** | **Diet** | **Alcohol** |
| --- | --- | --- | --- |
| **Model 1** | **n=2,583** | **n=2,551** | **n=1,857** |
| Psychological distress | 1.13 (.99-1.28) | 1.02 (.84-1.23) | 1.68 (1.33-2.12)* |
| Sex | 1.04 (.78-1.39) | .88 (.58-1.34) | .92 (.50-1.70) |
| Psychological distress x sex | 1.17 (.99-1.38)^a^ | 1.30 (1.03-1.65)^a^ | .94 (.69-1.27) |
| Age | 1.01 (1.00-1.01) | .99 (.98-1.00)* | .98 (.96-.99)* |
| Clinical anxiety | .97 (.72-1.31) | 1.03 (.68-1.56) | 1.32 (.80-2.16) |
| Clinical depression | 1.11 (.80-1.54) | 1.08 (.68-1.71) | .95 (.54-1.65) |
| Wave 2 | .09 (.70-1.03) | .95 (.71-1.26) | .97 (.65-1.46) |
| Wave 3 | .49 (.40-.60)* | .82 (.61-1.09) | 1.65 (.16-2.34)* |
| **Model 2** | **n=2,607** | **n=2,570** | **n=1,873** |
| Worry | 1.23 (1.01-1.49) | 1.11 (.84-1.48) | 1.28 (.91-1.80) |
| Sex | 1.01 (.51-1.98) | .87 (.33-2.32) | 1.30 (.40-4.28) |
| Worry x sex | 1.11 (.85-1.44) | 1.17 (.80-1.71) | .85 (.54-1.35) |
| Age | 1.00 (1.00-1.01) | .98 (.98-.99)* | .97 (.96-.98)* |
| Clinical anxiety | 1.09 (.81-1.47) | 1.16 (.76-1.75) | 1.69 (1.05-2.74)* |
| Clinical depression | 1.16 (.83-1.60) | 1.11 (.70-1.76) | 1.02 (.59-1.76) |
| Wave 2 | .86 (.71-1.05) | .95 (.72-1.26) | .95 (.64-1.42) |
| Wave 3 | .50 (.41-.61)* | .82 (.62-1.10) | 1.65 (1.16-2.33)* |
| **Model 3** | **n=2,610** | **n=2,573** | **n=1,874** |
| Sex | 1.34 (1.07-1.66)* | 1.32 (1.04-1.68)* | .88 (.66-1.19) |
| Age | 1.00 (1.00-1.01) | .99 (.98-.99)* | .97 (.96-.98)* |
| Clinical anxiety | 1.12 (.76-1.65) | 1.18 (.78-1.77) | 1.72 (1.07-2.78)* |
| Clinical depression | 1.17 (.76-1.79) | 1.13 (.72-1.79) | 1.03 (.59-1.77) |
| Wave 2 | .83 (.64-1.06) | .93 (.70-1.23) | .92 (.62-1.37) |
| Wave 3 | .48 (.37-.63)* | .80 (.60-1.06) | 1.61 (1.14-2.28)* |
