# Supplementary material for: How sex impacted associations between psychological distress and worry on adults’ health behaviours during SARS-CoV-2
Source: PLoS One. 2025 Dec 29;20(12):e0339274. doi: 10.1371/journal.pone.0339274 (PMC12747350; doi:10.1371/journal.pone.0339274)
Supplement: S4 Table — Odd ratios and 99% confidence intervals shown. Reference group for sex (male), clinical anxiety (no diagnosis), clinical depression (no diagnosis), wave (2). n = number. *p < .01. amoderator p-value < .10. Interaction term kept in multivariable model. (DOCX) [file pone.0339274.s004.docx]

| **Ireland** | **Physical activity** | **Diet** | **Alcohol** |
| --- | --- | --- | --- |
| **Model 1** | **n=2,889** | **n=2,912** | **n=2,324** |
| Psychological distress | 1.60 (1.35-1.88)* | 1.81 (1.49-2.19)* | 1.64 (1.36-1.97)* |
| Sex | .998 (.62-1.58) | 1.61 (.94-2.75) | .90 (.51-1.58) |
| Psychological distress x sex | 1.04 (.82-1.31) | .84 (.64-1.08)^a^ | 1.00 (.76-1.32) |
| Age | .99 (.98-1.00) | .98 (.97-.99)* | .99 (.98-1.00)* |
| Clinical anxiety | 1.32 (.95-1.83) | 1.54 (1.10-2.16)* | 1.22 (.84-1.77) |
| Clinical depression | .85 (.58-1.25) | .80 (.53-1.19) | .88 (.56-1.36) |
| Wave 3 | .96 (.71-1.29) | .83 (.60-1.14) | .98 (.70-1.37) |
| Wave 4 | 1.16 (.86-1.56) | .94 (.68-1.30) | .83 (.59-1.16) |
| Wave 5 | .98 (.72-1.33) | .90 (.65-1.25) | .78 (.55-1.11) |
| **Model 2** | **n=2,895** | **n=2,918** | **n=2,326** |
| Worry | 1.17 (.97-1.41) | 1.04 (.85-1.28) | 1.00 (.82-1.22) |
| Sex | 1.10 (.58-2.09) | 1.07 (.54-2.12) | .80 (.34-1.68) |
| Worry x sex | 1.01 (.77-1.33) | 1.07 (.80-1.44) | 1.12 (.82-1.53) |
| Age | .98 (.98-.99)* | .97 (.96-98)* | .98 (.97-.99)* |
| Clinical anxiety | 1.62 (1.18-2.22)* | 1.87 (1.34-2.61)* | 1.52 (1.05-2.19)* |
| Clinical depression | 1.01 (.69-1.48) | 1.87 (1.34-2.61)* | 1.08 (.70-1.66) |
| Wave 3 | .93 (.69-1.25) | .80 (.58-1.09) | .94 (.68-1.30) |
| Wave 4 | 1.07 (.80-1.44) | .88 (.64-1.21) | .78 (.55-1.07) |
| Wave 5 | .95 (.33-1.28) | .87 (.55-1.90) | .76 (.53-1.07) |
| **Model 3** | **n=2,900** | **n=2,923** | **n=2,331** |
| Sex | 1.16 (.93-1.45) | 1.27 (1.00-1.61) | 1.03 (.80-1.33) |
| Age | .99 (.98-.99)* | .97 (.96-.98)* | .98 (.97-.99)* |
| Clinical anxiety | 1.66 (1.21-2.80)* | 1.89 (1.36-2.64)* | 1.53 (.106-2.20)* |
| Clinical depression | 1.02 (.70-1.49) | .96 (.65-1.43) | 1.08 (.70-1.66) |
| Wave 3 | .90 (.67-1.21) | .78 (.57-1.07) | .93 (.67-1.29) |
| Wave 4 | 1.08 (.81-1.44) | .88 (.64-1.20) | .77 (.55-1.08) |
| Wave 5 | .94 (.48-1.20) | .86 (.62-1.19) | .75 (.53-1.07) |
