# Supplementary material for: How sex impacted associations between psychological distress and worry on adults’ health behaviours during SARS-CoV-2
Source: PLoS One. 2025 Dec 29;20(12):e0339274. doi: 10.1371/journal.pone.0339274 (PMC12747350; doi:10.1371/journal.pone.0339274)
Supplement: Attachment — Submitted filename: Response to Reviewers.docx [file pone.0339274.s006.docx]

We thank the reviewers and editor for their time and thoughtful feedback when reviewing this manuscript. Below we have outlined all queries and your responses in italics. Unmarked and marked up versions of the revised manuscript have been attached as separate documents in the online portal. Line numbers referenced below correspond to the marked up version in the portal (not the unmarked version).

Journal feedback:

1. Please ensure that your manuscript meets PLOS ONE's style requirements, including those for file naming. The PLOS ONE style templates can be found at:

<https://journals.plos.org/plosone/s/file?id=wjVg/PLOSOne_formatting_sample_main_body.pdf>

[https://journals.plos.org/plosone/s/file?id=ba62/PLOSOne_formatting_sample_title_authors_affiliations.pdf](https://track.editorialmanager.com/CL0/https:%2F%2Fjournals.plos.org%2Fplosone%2Fs%2Ffile%3Fid=ba62%2FPLOSOne_formatting_sample_title_authors_affiliations.pdf/1/010f0198f065b24e-b62a9b7d-23d2-4219-9cd2-9a97172fd6ed-000000/PGt3dgP20XzrgFA0wCDbU9X5iCsjglDGF5LmXHA58lY=226)

*We have reviewed the formatting documents and changed the file naming as indicated.*

2. Thank you for stating the following financial disclosure:
   This iCARE study was supported by the Canadian Institutes of Health Research (CIHR: MM1-174903; MS3-173099; SMC- 151518); the Medical Science Fonds of the Major of Vienna (COVID020); the Canada Research Chairs Program (950- 232522, Chair holder: Dr. Kim L. Lavoie); the Fonds de recherche du Québec—santé (FRQ-S: 251618; 34757); the Fonds de recherche du Québec société et culture (FRQSC: 2019-SE1-252541); and the Ministère de l’ Économie et de l’Innovation du Québec (2020-2022-COVID-19-PSOv2a- 51754). Study sponsors had no role in conducting the research. None of the funders were involved in the study design or activities of this manuscript. Please state what role the funders took in the study. If the funders had no role, please state: "The funders had no role in study design, data collection and analysis, decision to publish, or preparation of the manuscript."
   If this statement is not correct you must amend it as needed.
   Please include this amended Role of Funder statement in your cover letter; we will change the online submission form on your behalf.

*The funders had no role in the study design, data collection and analysis, decision to publish or preparation of the manuscript. We have amended the text to include this statement in the cover letter.*

3. Thank you for stating the following in the Competing Interests/Financial Disclosure section:
   Two authors (KL and SB) were included in the set-up of iCARE and management its current activities. All other authors have no financial or conflicts of interest to declare. We note that one or more of the authors are employed by a commercial company: iCARE. Please provide an amended Funding Statement declaring this commercial affiliation, as well as a statement regarding the Role of Funders in your study. If the funding organization did not play a role in the study design, data collection and analysis, decision to publish, or preparation of the manuscript and only provided financial support in the form of authors' salaries and/or research materials, please review your statements relating to the author contributions, and ensure you have specifically and accurately indicated the role(s) that these authors had in your study. You can update author roles in the Author Contributions section of the online submission form.

*iCARE is not a commercial company. iCARE is an international research study that represents a collaboration between multiple international research teams. The project is led out of the Montreal Behavioural Medicine Centre by Drs. Lavoie and Bacon. As such, iCARE does not have a funding role it is merely the name of an international research study led by the Canadian researchers.*

4. Please also include the following statement within your amended Funding Statement. “The funder provided support in the form of salaries for authors [insert relevant initials], but did not have any additional role in the study design, data collection and analysis, decision to publish, or preparation of the manuscript. The specific roles of these authors are articulated in the ‘author contributions’ section.”
   If your commercial affiliation did play a role in your study, please state and explain this role within your updated Funding Statement.

*None of the funders provided salary support for any of the authors. Instead, funding bodies listed provided operational funds for the research to be conducted. For example, contributing funds to pay research staff that assisted with data collection and participant incentives.*

5. Please also provide an updated Competing Interests Statement declaring this commercial affiliation along with any other relevant declarations relating to employment, consultancy, patents, products in development, or marketed products, etc. Within your Competing Interests Statement, please confirm that this commercial affiliation does not alter your adherence to all PLOS ONE policies on sharing data and materials by including the following statement: "This does not alter our adherence to PLOS ONE policies on sharing data and materials.” (as detailed online in our guide for authors <http://journals.plos.org/plosone/s/competing-interests>) . If this adherence statement is not accurate and there are restrictions on sharing of data and/or materials, please state these. Please note that we cannot proceed with consideration of your article until this information has been declared.

*Again, iCARE is not a commercial company. It does not produce profit, nor operate as a business. It is the name of an international research study.* *As such, there is no violation to PLOS ONE policies on sharing data and materials.*

6. Please include both an updated Funding Statement and Competing Interests Statement in your cover letter. We will change the online submission form on your behalf.

*The updated Funding Statement as indicated in query 2 above. As there are no changes to the authors competing interests as iCARE is not a commercial company, nor does it pay any of the co-authors listed here, the Competing Interests Statement remains the same.*

7. One of the noted authors is a group or consortium The iCARE Study team. In addition to naming the author group, please list the individual authors and affiliations within this group in the acknowledgments section of your manuscript. Please also indicate clearly a lead author for this group along with a contact email address.

*The iCARE project team encompasses staff and trainees who assist with daily research project tasks of the broader iCARE study team. This list changes monthly based on student, trainee, volunteer and staff changes. As such, it is not possible to produce a list of all the individuals involved on the iCARE study team. There is also no lead author for the iCARE study beyond Drs. Lavoie and Bacon, both Drs. Lavoie and Bacon are the leaders of the entire iCARE study team. This text has been added to the acknowledgements section of the manuscript along with the general project email address. We have also added the Montreal Behavioural Medicine Centre (MBMC) affiliation to the iCARE Study team to make the connections clearer to a reader.*

8. In the online submission form, you indicated that this is secondary analysis of cross-sectional data collected as a part of the International COVID-19 Awareness and Responses Evaluation (iCARE) Study led by the Montreal Behavioural Medicine Centre (MBMC; a joint Centre intégré universitaire de santé et de services sociaux du Nord-de-l’Île-de-Montréal (CIUSSS- NIM)/Université du Québec à Montréal/Concordia University academic research and training centre). All data available by request from the [www.icarestudy.com](http://www.icarestudy.com) website. All PLOS journals now require all data underlying the findings described in their manuscript to be freely available to other researchers, either a. In a public repository, b. Within the manuscript itself, or c. Uploaded as supplementary information. This policy applies to all data except where public deposition would breach compliance with the protocol approved by your research ethics board. If your data cannot be made publicly available for ethical or legal reasons (e.g., public availability would compromise patient privacy), please explain your reasons on resubmission and your exemption request will be escalated for approval.

*As this is a secondary data analysis, the original ethics for the iCARE project have outlined that the data is stored and administered through the iCARE Study online repository. All data we used can be provided by the iCARE study team through a request to made on the iCARE website indicated. The iCARE Study team manages all project data securely on their servers at the MBMC as a public repository – anyone can request access to the data via the online repository the iCARE Team manages. As these are the procedures in place for the iCARE study itself, we are unable to alter them as we are merely using the freely available data provided by the iCARE Study team. Based on feedback of reviewers 1,2 and 3 in the “Have the authors made all data underlying the findings in their manuscript fully available?” question all responding “yes,” we believe that it is clear to the reader that all data we used can be publicly accessed for free.*

9. Please remove all personal information, ensure that the data shared are in accordance with participant consent, and re-upload a fully anonymized data set.

Note: spreadsheet columns with personal information must be removed and not hidden as all hidden columns will appear in the published file.

Additional guidance on preparing raw data for publication can be found in our Data Policy (<https://journals.plos.org/plosone/s/data-availability#loc-human-research-participant-data-and-other-sensitive-data>) and in the following article: [http://www.bmj.com/content/340/bmj.c181.long](http://track.editorialmanager.com/CL0/http:%2F%2Fwww.bmj.com%2Fcontent%2F340%2Fbmj.c181.long/1/010f0198f065b24e-b62a9b7d-23d2-4219-9cd2-9a97172fd6ed-000000/sks-wDpoILGtT8nOibusqA3waWAOUghzRXiheUVmRYU=226).

*None of the uploaded data we have provided in tables or figures contains personal information. No columns are hidden. If the journal can confirm where they have seen personal information in the data presented, we are more than happy to ensure it is appropriately removed.*

10. If the reviewer comments include a recommendation to cite specific previously published works, please review and evaluate these publications to determine whether they are relevant and should be cited. There is no requirement to cite these works unless the editor has indicated otherwise.

*Three studies that have previously been published were suggested by reviewer 3. We have carefully reviewed these studies and included one of the suggested ones in the revised manuscript.*

Reviewer #1 comments:

1. In the abstract, you should provide full name of COVID-19.

*The full name of severe acute respiratory syndrome coronavirus 2* *has been added in the abstract and abbreviated as SARS-CoV-2.*

2. Please provide full name of OR.

*The full name of ‘odds ratio’ has been added.*

3. You need to give an example of the tailored program.

*An example has been written in text as “public health campaign promoting culturally relevant physical activity in response to greater perceived psychological distress.” We have also specified that we are talking about a tailored behavioural intervention.*

4. I just wonder, why you used P<.1 to put variables into the equation and <0.1 for the significant level, normally we used 25%.

*We believe the second p-value here above may be a typo. We used p<.10 to explore if an individual independent variable (i.e., psychological distress and infection worry) and sex warranted inclusion in the final model with both independent variables present. This process of conservative checking is one that is used in model building in a variety of settings and is more conservative than using .25 (See example reference below). In the final model with both psychological distress and infection worry we used a p-value <.01 instead of the typical p<.05. In both cases we chose to use more conservative values as we run a large number of models and aimed to reduce the likelihood of spontaneous significance from arising in our findings.*

*Deslippe AL, González OD, Buckler EJ, Ball GDC, Ho J, Bucholz A, Morrison KM, Mâsse LC. Do Individual Characteristics and Social Support Increase Children's Use of an MHealth Intervention? Findings from the Evaluation of a Behavior Change MHealth App, Aim2Be. Child Obes. 2023 Oct;19(7):435-442. doi: 10.1089/chi.2022.0055. Epub 2022 Dec 22. PMID: 36576875.*

Reviewer #2 comments:

1. Please consider revising the title to make it more concise.

*We have shortened the title to “How sex impacted associations between psychological distress and worry on adults’ health behaviours during SARS-CoV-2.”*

2. The abstract effectively sets the context and highlights key findings of the study, but a few improvements can enhance clarity including: Consider making the abstract short and precise as it is written very long, clarify the implications of sex as a moderator in the results, ensure consistent terminology and keep statements concise for better readability.

*We have shortened the text in the abstract from the maximum word count of 300 to 270. We have clarified the meaning of sex in the context of moderation in Columbia (“…with females experiences more delirious changes (p<.01)”) and replaced terminology throughout to repeat the same words.*

3. The introduction effectively outlines the impact of COVID-19. However, consider clarifying some points for better flow and coherence. For instance, provide clearer transitions to enhance readability when mentioning studies and ensure that the rationale for the country-level comparison is clearly articulated, emphasizing its relevance to the study's objectives.

*We have altered wording throughout the introduction to make it clearer when a previously published study is being discussed and specified the country of each study to emphasize diversity in trends across countries. We have also adapted the wording in lines 818-826 to re-iterate why country comparisons are useful to establish sex-based trends (as opposed the gender-based trends that can impact health behaviours).*

4. Specify the criteria for compensation to ensure transparency when describing participant recruitment.

*We have altered the wording in lines 870-871to emphasis the individual approach that each polling service in the respective countries employed across the iCARE study.*

5. The eligibility criteria could be simplified for better readability.

*We have simplified the wording to:*

*To be included in this analysis, participants from Canada, Columbia and Ireland who completed an online survey from April 2020-December 2022 who reported changes in their health behaviours “since the start of COVID-19 (referring to SARS-CoV-2)” were assessed in this analysis. (Line 886-888)*

6. Clarify how the internal consistency of the infection worry variable was assessed

*We had used and presented Pearson correlations of each of the four items assessing worry in a pairwise fashion. We used a cut off of .80 to indicate high correlation and inappropriateness of creating a summary worry variable. Correlations were assessed in each country. We have adapted the metric to present Cronbach Alpha’s instead now as this is a more typical and recognized metric that can be used to indicate summary variable reliability (lines 931-933).*

7. How are the statistical analysis methods clearly linked to the research questions?

*We have revised the Data Analysis section to make the links between what was done and the research question clearer.*

8. The results section effectively presents key findings across the three countries, highlighting significant differences in health behaviors related to psychological distress and infection worry. However, consider providing clearer summaries of the statistical findings for each country to enhance readability. For instance, clarify the impact of demographic factors, such as age and clinical diagnoses, on the results to contextualize the findings better.

*Given that clarifying the impacts of demographic factors, such as age, on the result to contextualize them beyond present the values themselves (as shown) would enter more into a discussion space we have not incorporate this suggestion in the results section. We have however, articulated the impact of co-variates more clearly in the discussion section (see lines 1182-1217).*

9. It may also be helpful to include a brief discussion on the significance of the observed sex differences in health behaviors to tie the results back to the study's main objectives.

*This has been incorporated with the query below (#10) as a discussion sub-section summary (lines 1182-1217).*

10. Consider enhancing the clarity of the arguments by summarizing key findings more succinctly.

*We have added in summaries at the end of the main discussion paragraphs (lines 1128-1132, lines 1191-1207 and lines 1216-1217).*

11. Additionally, while the limitations are well-articulated, it may strengthen the section to emphasize the implications of self-report bias and the need for future longitudinal studies.

*This has been added to lines 1224-1227:*

*These limitations make it imperative for future research that relies on more objective measurement of changes in mental well-being and health behaviours (e.g., cross-references to medical charts for incidence of new health conditions arising during and shortly after SARS-CoV-2). It is also critical that longitudinal research exploring the prolonged impacts of SARS-CoV-2 are conducted to provide a clearer picture of long-term impacts as health behaviours do not instantaneously alter health outcomes, and instead, maintained patterns do over prolonged periods of time.*

12. The conclusions effectively summarize the study's findings and emphasize the significant impact of COVID-19 on mental well-being and health behaviors. Consider refining the wording for greater clarity, particularly in the call for country-specific interventions. It may be beneficial to explicitly state the implications of the findings for public health policy in each country.

*We added an example of what a possible public health effort that could apply across countries and what factors may be important to consider within countries to tailor a behavioural intervention (lines 1249-1254).*

13. Additionally, while the focus on reducing psychological distress is important, expanding on potential strategies for implementing these interventions could enhance the conclusion's practical relevance.

*We expanded on this with an example strategy in lines 1251-1254.*

14. Lastly, reiterating the importance of understanding sex differences in health behaviors could further strengthen the argument for targeted approaches in future pandemics.

*We have re-iterated the important role of sex in mental well-being across countries (1254-1323).*

Reviewer #3 comments:

I have many issues concerning the (a) write-up of the manuscript, and (b) the method / results employed by the authors. Given the database the authors are drawing on, I hope the comments and examples given below can further the inherent value of this science and author’s revision of the manuscript.

15. Line 51: Your first sentence - “Coronavirus disease or COVID-19 is an infectious disease…” is filled with a possible redundancy, ambiguity and inaccuracy. When using “Coronavirus disease” which one are you refereeing to? In terms of the recent pandemic the viral infection is referred to as SARS-CoV-2. The “COVID-19” moniker refers to the pandemic event; this is a difference between the event and the infection. Please write a direct sentence, avoid the use of the little word “or”; the sentence reads like a multiple-choice question; either coronavirus disease or COVID-19 is an infection disease. Please be direct, precise and accurate throughout the manuscript.

*We had elected to go with the more lay term (i.e., COVID-19) as it was used in all questions and procedures of the iCARE study, and has been found to be identifiable by the public. We appreciate that this is not a technically clear name and as such, have changed COVID-19 to SARS-CoV-2 in manuscript text. Any instances where COVID-19 was the published terminology used, such as in all iCARE questions referenced, we have left the text as COVID-19 to accurately reflect wording that was used.*

16. Line 66: The authors said, “From a psychological standpoint” anxiety symptom “or” depression are linked to impaired “or” poorer health. Again, please reconsider your literature review write up, and take care in the use of “or”. Using psychology as a “standpoint” appears too general; there are many psychological approaches and fields, - you should be more precise; direct the reader’s understanding to a specific approach or field of psychology concerning anxiety and health.

*We removed the wording of “from a psychological standpoint.” We have amended wording throughout the introduction to streamline the writing and replaced the word ‘or’ with ‘and’ when two separate concepts are discussed.*

17. Line 84: Authors write “Sex is rooted in biological mechanisms, and in the context of COVID-19, appears to influence trends between psychological distress….” Given my read of the overall manuscript, the importance of this paragraph is critical to the logic, analysis, and conclusions of this research. Using biological mechanism as a pretext for understanding sex-differences in the context of COVID-19 is a misapplied concept. Yes, sex determination as being a biological mechanism (e.g., genetic and epigenetic processes) I have no doubts. But the authors have not included a literature review where sex-differences are associated with socialization, gender development, stereotyping, culturalization that differentiate male/female preferences, expectations, and behaviors. Starting on Line 86, with a discussion about Canada, France, United Kingdom and Zimbabwe, are general descriptions reporting how different sexes have responded differently and have responded differently across differently countries. It is difficult to understand the logic of the authors how the biological mechanism alone (and not a social mechanism) becomes the explanation (i.e., sex as an important moderator – as claimed in the manuscript).

*This concern is exactly why we have conducted this analysis. In many studies sex and gender are used interchangeably, despite the fact that they are unique constructs. Give that sex is often measured most in research and applied in comparison to gender, we aimed to call specific attention as to how this may be (in)appropriate in the context of behavioural interventions during a pandemic. As you have suggested, gender is likely the more salient factor to consider in the discussion of physical activity and dietary behaviours as it captures social pressure and norms. To help make this clearer we have adjusted wording throughout the introduction and discussion to re-iterate how sex, a biological factor, is different from gender as a social construct, and likely not as salient to consider in behavioural interventions seeking to support adults practice of health behaviours given the evidence at this time. We are not suggesting that biological sex will be the only factor impacting changes in mental well-being and health behaviours. Instead, we are suggesting that it is one important factor and is not to be conflated with social factors like gender; sex should be explored in its own right to truly understand how best to tailor any behavioural intervention.*

18. There is no review of sex differences rooted in a social/societal mechanism (e.g., see Social Psychology literature) in the introduction. Because the authors have conceptually focused on the sex-based factor in the write up and analyses, I strongly recommend the authors more thoroughly review and discuss sex-difference literature, especially concerning how sex-differences are understood socially and psychologically (i.e., sex-based preferences and expectations). As is, the authors have dedicated one paragraph to what is a central theoretical explanation to their results and their write up. Please make note of the sub-title of the manuscript, “…: A Sex-Based Analysis”.

*This approach is intentional. We have elected to focus on sex as the biological variable that it is and not inherently focus on sex as an interconnected facet of gender identity (a social construct). We do appreciate that sex and gender do not exist in isolation from one another, and neither exists in a vacuum. To help make the distinction and interconnections clear between sex and gender we have added lines 701-709, 754-755 and 1067-1070). As noted, we conducted a sex-based analysis and not a gender-based analysis, and it is our intent to make this difference clear as these unique constructs should not be conflated with one another.*

19. Line 97: Authors begin a review of “country-level comparisons” and claim country-level comparisons “are effective method to identify consistent patterns in how sex may influence the associations between …” The authors go on to claim “This is because sex is based on biological factors that transcend geographical or cultural boarders”. Is there not a subtle contradiction implied here between these two contiguous sentences? Country-level comparisons are important to identify how sex influences, but then sex identified as a biological factor transcends the country-level factor? I do note the word difference and word substitution.

*We are unsure what the concern is with the wording used in these sentences. If the reviewer can clarify what the specific concern (or change they are hoping to see is) we would be happy to amend accordingly for clarity.*

20. Never-the-less, there is little literature review regarding the importance of cultural / national influences on people’s habits and health practices. The authors again appear to neglect the psychological, sociological and cultural approaches to better understand “consistent” and inconsistent patterns among people. Again, I recommend authors to seek the literature, maybe starting in general with Harry Triandis’s psychological approach and what he called Cultural Syndromes. Yes, teasing out theoretically socially rooted sex-differences, and the connection with cultural differences is a challenge. But once again, this manuscript has focused on “country-level comparisons” and has dedicated very little literature review in the introduction on this topic.

*It is beyond the scope of this analysis to delve into social constructs such as culture and gender norms. We completely agree that these factors are critical in shaping human behaviours, but it is not within the aim of this analysis to delve into. Our aim is to “clarify how sex may affect the associations between changes in mental well-being and health behaviours”. Thus, to ensure sex is not conflated with social based factors, it is important that we focus the content of this analysis on sex. We are in full support of future literature untangling the interconnections between sex and social factors, like gender, but we do not have the data collected here to draw or speak to those conclusions.*

21. Line 106: Authors describe the method in general as a secondary analysis of cross-sectional data. This data is a part of a greater data-base, specified as iCARE and led by MBMC. On Line 118, the authors briefly describe the data base as encompassing 42 countries. The authors then describe the different waves of recruitment. And then on Lines 128-129 the authors identify three nations to be analyzed for this manuscript: Canada, Columbia, and Ireland. I recommend that the authors explain the procedure for how these three nations were selected from the 42 counties listed in the iCARE database. No mention of this potential selection bias was mentioned – nor discussed in the limitation section of the Discussion. I refer the authors to the Clark et al. reference (see below) and take special note of the section 2.11 on p. 77. This section might be a good model for writing up a revision of this procedure.

Clark C, Davila A, Regis M, Kraus S. Predictors of COVID-19 voluntary compliance behaviors: An international investigation. Glob Transit. 2020;2:76-82. doi: 10.1016/j.glt.2020.06.003. Epub 2020 Jun 26. PMID: 32835202; PMCID: PMC7318969.
(See p 77, Section 2.11 – the Open Science Statement)

*This rational is present in lines 771-792. We have also added in reference to the Clark et al. paper in the strengths section of the discussion.*

22. Line 187: Section on Data Analysis describes the “exploration” of data by first seeking difference (“t-test and chi-square tests”) between selected countries (i.e., Canada, Columbia, and Ireland). The authors describe using logistic regression as means to isolate significant relations between variables for each country separately. Thus, I assume three different analyses, that is three different logistic regressions (Tables S2, S3, & S4). I realize the authors have identified the methodology as exploratory, but I question why the authors did not build and test an omnibus statistic or model to incorporate a country-level factor with the inclusion of the sex factor? I believe the paper’s logic and strength of contribution becomes compromised throughout by not clearly providing and structuring this overall analysis.

*As each country has incredibly unique facets that were not collected within the scope of this dataset, we believe that it is most appropriate to conduct the stratified analysis based on country. In this way, we can observe trends in the associations between measures of mental well-being and health behaviours in their respective context. Our approach still facilitates broader conclusions across countries in comparison to one another. Combing the data into one dataset may not be an appropriate approach as the exact wording on a country’s questionnaire was not 100% the same given local language and context. Using a regression model with country as a confounder would also only allow us to compare effectively to a reference country, and not necessarily as fluidly across the three.*

23. Line 285: A typo in the main heading – “Psychological distress had a consist role in …” – the word “consist” should read “consistent”.

*We have fixed the typo to read ‘consistent.’*

24. One of the main goals of science is to identify and test variables for the purpose of (a) prediction and (b) treatment/intervention. The many nuanced findings in this manuscript (including similarities and differences) between country-level comparison makes for a difficult interpretation of the variables tested. See Line 337, the authors provide their take on the paper’s strengths. Basically, the strength of the paper is - you can “do” this research. Yes, you can “do” this research – but what did this paper actually tell us about the sex-based factor and the country-level factors? The authors statement - “sex-based trends can be assessed regardless of country-level factors” seems to be an overstatement without evidence provided by the author’s research. Actually, many of the statements given in the manuscript provide evidence concerning the importance of the country-level factor. For instance, reading over the papers many diverse and singular findings, simply stated here: (a) you show how Canada and Ireland show consistent relations, while Columbia does not, or (b) Canada and Columbia show consistent relations, but not Ireland. Of course, adding sex-factor moderator increases the interpretative nuance. But as an applied health care expert, how do I “use” this information provided in this manuscript? I believe a revision of this manuscript is necessary to clarify the logic, statistical analysis, and the contribution of the findings; that is, how do these variables predict (or do not predict) and how do we then treat the negative effects of a future pandemic (is it at the sex-based factor, country/cultural factor, regime-based factor… etc).

*We have added examples of how the data we have presented can be used more explicitly within the healthcare sector. We have also re-iterated how the exploratory findings are critical to understanding behavioural mechanisms that are imperative to shape the development of any future behaviour intervention in a future pandemic. This time of exploration is the first step, the ‘basic behavioural science phase’ where critical targets of a future intervention can begin to be understood. As an example of this process and why it is critical to start with behavioural mechanism exploration see:*

*Michie S, Marques MM, Norris E. Theories and Interventions in health behaviour change. Handb Health Psychol. Published online 2019:69-88.*

*Czajkowski SM, Powell LH, Adler N, et al. From ideas to efficacy: The ORBIT model for developing behavioral treatments for chronic diseases. Health Psychol. 2015;34(10):971-982. doi:10.1037/hea000016*

25. Also, these references might be helpful toward revising the logic and method of this manuscript.

    Hensel L, Witte M, Caria AS, Fetzer T, Fiorin S, Götz FM, Gomez M, Haushofer J, Ivchenko A, Kraft-Todd G, Reutskaja E, Roth C, Yoeli E, Jachimowicz JM. Global Behaviors, Perceptions, and the Emergence of Social Norms at the Onset of the COVID-19 Pandemic. J Econ Behav Organ. 2022 Jan;193:473-496. doi: 10.1016/j.jebo.2021.11.015. Epub 2021 Nov 19. PMID: 34955573; PMCID: PMC8684329.

    He, Z., Jiang, Y., Chakraborti, R., & Berry, T. D. (2022). The Impact of National Culture on Covid-19 Pandemic Outcomes. Internatinal Journal of Social Economics., 49(3), 313-335 <https://doi.org/10.1108/IJSE-07-2021-0424>

*We thank the reviewer for these interesting manuscripts focusing on social factors that impacted behaviours during the pandemic. We will keep these in mind for our future research that uses a social lens for behaviour change (e.g., exploration of cultural and gender norms on behaviours), opposed to the lens applied here focused on biological factors.*
