# Supplementary material for: How sex impacted associations between psychological distress and worry on adults’ health behaviours during SARS-CoV-2
Source: PLoS One. 2025 Dec 29;20(12):e0339274. doi: 10.1371/journal.pone.0339274 (PMC12747350; doi:10.1371/journal.pone.0339274)
Supplement: Attachment — Submitted filename: Response_to_Reviewers_auresp_2.docx [file pone.0339274.s007.docx]

We thank the reviewers and editor for their time reviewing our revisions to this manuscript. Below we have outlined all queries and our responses in italics. Unmarked and marked up versions of the revised manuscript have been attached as separate documents in the online portal. Line numbers referenced below correspond to the marked-up version in the portal (not the unmarked version).

Journal feedback:

1. If the reviewer comments include a recommendation to cite specific previously published works, please review and evaluate these publications to determine whether they are relevant and should be cited. There is no requirement to cite these works unless the editor has indicated otherwise.

*Three studies that have previously been published were suggested by reviewer 3. We have carefully reviewed these studies and included one of the suggested ones in the revised manuscript.*

2. Please review your reference list to ensure that it is complete and correct. If you have cited papers that have been retracted, please include the rationale for doing so in the manuscript text, or remove these references and replace them with relevant current references. Any changes to the reference list should be mentioned in the rebuttal letter that accompanies your revised manuscript. If you need to cite a retracted article, indicate the article’s retracted status in the References list and also include a citation and full reference for the retraction notice.

*We have reviewed our reference list and all references are accurate. We have not included any redacted papers in our reference list to the extent of our knowledge.*

Reviewer #3 comments:
After reading over the three reviewers’ comments on the original manuscript, I am impressed with authors efforts, edits, and the changes made to the original manuscript. The revised manuscript is a much better write-up.

1. My only reservation involves the how the three nations were selected (i.e., Canada, Columbia and Ireland). I wonder if the authors could clarify how these three nations were selected out of the 42 nations that completed (or nearly completed) the electronic surveys. I note the Gunther Eysenbach citation, and understand the use of CHERRIES. I note that the three nations had good completeness scores (98%, 95.5% and 78.7%). With that being noted, how again were Canada, Columbia and Ireland selected from the original 42 nations? For instance, were these nations selected because they represented the “top three” completeness scores, thus more statistically stable than the other 39 other nations?

   Maybe you could include the mean and ranges of the completeness score of all 42 nations (as you would in a basic Method section under a Participant section description of a human participant sample).

   For example, (“… the average completeness score of the 42 nations was 50%, ranging from 23% to 98%.”). Again, if I am missing something here, I apologize; but I do not read in your revision, how Canada, Columbia and Ireland were specifically selected out of the 42 nations that had some chance or opportunity to be included in your analysis.

   Again, were the three nations selected because they were the top three? Or was another consideration used for inclusion. Please note the difference between stating “Eligibility for this analysis” (line 156) and “Inclusion for this analysis”.

   The manuscript notes eligibility as defined as completeness (connection to Eysenbach), but the paper does not mention the overall baseline or a comparison score to the other less eligible nations (the other 39 countries). Of course, this the asks the question, how was inclusion (selection) of a nation/country defined? How was this inclusion unbiased, and unbiased given the completeness scores of the other 39 nations?

*Selected of Canada, Ireland and Columbia had nothing to do with their completion rate. We present the completion rate as it is important to report clearly to increase the transparency of data reporting using cross-sectional studies (see the CHERRIES checklist for reference to this criteria).*

*As we indicated on line 134-136 “Canada, Columbia and Ireland were selected for data analysis as these three countries had complete nationally representative data collected for questions relevant to measures assessing changes in mental well-being and health behaviours attributed to SARS-CoV-2.”*

*We can appreciate that this may still be confusing as to why the three countries were chosen. First, these three countries had nationally representative data samples recruited based on demographic factors – not all of the other countries that participated in the iCARE study had this. To help minimize responder bias between countries based on a demographic confounder we believe it is important to use nationally representative data to get a clearer picture based on individual country context. Only Australia, Canada, Colombia, France, Ireland, Israel, Italy, the United Kingdom and the United States collected representative data and would fit this.*

*The second important factor was that these three countries had the same wording in how changes to health behaviours was asked. Not all countries collected the exact same data, and not all countries used the same wording in their surveys. This was done to give researchers of different countries some autonomy to tailor the cross-sectional surveys to respond to information that their own health officials wanted to have collected.*

*The third important factor is that these three countries specifically had their waves of data collection completed and cleaned. The iCARE team had a few other countries that were actively still recruited data using the same type of questions of nationally representative samples, but these were not finished collection and thus, not available to use yet (e.g., Italy and France). When these countries are fully collected and cleaned it would be useful to consider the methods of this analysis in those countries to see if trends we identified in Canada, Columbia and Ireland prevail. To re-iterate the multi-faceted nature of country selected for our analysis we have expanded the text to include the additional information (Line 133-145):*

*“Of the 42 countries that participated in the iCARE study, Australia, Canada, Colombia, France, Ireland, Israel, Italy, the United Kingdom and the United States collected data using representative samples of their respective populations based on demographic characteristics. To limit responder bias across countries on any potential confounding demographic factors, we explored data from nationally representative samples in the iCARE project that were fully collected, cleaned and asked about changes in mental well-being and health behaviours attributed to SARS-CoV-2. This left us with three countries that were eligible to use: Canada, Columbia and Ireland.”*

*We have also added the use of the three countries as a limitation to make it clear that we believe the analyses would have benefited from having included 42 countries – but it was not possible given how the data was collected (line 409-414):*

*“Finally, we were unable to explore data from all 42 countries in the iCARE Study due to differences in how samples were collected from diverse countries (i.e., nationally representative versus convenience), which questions were asked (i.e., countries could tailor their wording to national health aims) and which countries had their data fully collected and cleaned (i.e., not in progress). In would be incredibly insightful to explore trends across all 42 countries, had it been possible, to consider a more complete narrative of when trends may prevail and why.”*
